# TAK1 phosphorylation mediates macozinone (PBTZ169) induced innate immune activation against tuberculosis

**DOI:** 10.1128/msphere.00513-25

**Published:** 2025-09-22

**Authors:** Xinda Li, Xiaoyi Luo, Bin Wang, Lei Fu, Xi Chen, Yu Lu

**Affiliations:** 1 Department of Pharmacology, Beijing Chest Hospital, Capital Medical University 12517, Beijing, China; 2 Beijing Key Laboratory of Drug Resistance Tuberculosis Research, Beijing Tuberculosis and Thoracic Tumor Research Institute, Beijing, China; University of Nebraska Medical Center College of Medicine, Omaha, Nebraska, USA

**Keywords:** *Mycobacterium tuberculosis*, antitubercular drug, post-tuberculous lung disease, immunomodulation

## Abstract

The management of tuberculosis (TB), particularly drug-resistant variants, presents enduring clinical challenges characterized by complex therapeutic regimens, prolonged treatment durations, suboptimal success rates, and significant adverse effects, issues that have persisted as critical concerns in global healthcare. Current TB drug development predominantly focuses on novel compounds and combination therapies targeting pathogen-specific pathways while overlooking the influence of different drugs on host immunity, which is indeed a key factor affecting treatment-related tissue damage and treatment time. In this study, we evaluated the effects of important anti-TB drugs and candidate drugs on host innate immunity and found that PBTZ169 showed potent innate immunity activator, which is a promising drug for the treatment of drug-sensitive and -resistant TB. The expression of cytokines and type I interferon was strongly upregulated by PBTZ169 under lipopolysaccharide (LPS) stimulation and PBTZ169-resistant strain infection, and the innate immune activation enhanced antibacterial activity in macrophages. Mechanistically, PBTZ169 upregulated the NF-kB and MAPK signaling pathways by activating the phosphorylation of TAK1. TAK1 knockdown abrogated PBTZ169-mediated immune activation and antibacterial effects. We thus demonstrate for the first time that PBTZ169 up-regulates NF-κB and MAPK innate immune signaling pathways via activating TAK1 phosphorylation, which may inform clinical deployment strategies and patient selection.

**IMPORTANCE:**

Maintaining immune homeostasis is paramount for efficient *Mycobacterium tuberculosis* (Mtb) clearance and tissue repair. Current therapeutic strategies, however, predominantly focus on achieving maximal bacterial suppression within compressed timelines while overlooking the immunomodulatory consequences of anti-tuberculosis agents. This critical knowledge gap underscores the urgent need for mechanistic investigations to establish evidence-based frameworks for optimizing drug combinations and integrating therapies with host-directed approaches.

## INTRODUCTION

Tuberculosis (TB), a chronic infectious disease caused by *Mycobacterium tuberculosis* (Mtb) (1, 2), is again the leading single infectious cause of death in 2023, after being replaced by COVID-19 in 2020–2022. According to the epidemiological data of the World Health Organization, there were about 10.8 million new cases of TB globally in 2023, and the incidence continued to increase for 3 years. In addition, an estimated 25% of the global population has latent infection, which can progress to active TB when their immune status is compromised (3). It is worth noting that there were approximately 400,000 MDR-TB or RR-TB patients in 2023, further aggravating the difficulty of treatment. Current treatments for sensitive TB include isoniazid, rifampicin, pyrazinamide, and ethambutol, which usually require continuous treatment for 6 months (4–7). Treatment for drug-resistant TB requires the use of more second-line anti-TB drugs, such as bedaquiline, linezolid, pretomanid, clofazimine, and moxifloxacin, often for a longer period of treatment, up to 20 months (8, 9). To end the TB epidemic once and for all, we may need to study new strategies for TB treatment.

The maintenance of host immune homeostasis is essential for rapid eradication of pathogens, avoiding drug resistance, and reducing pathological damage to organs and tissues (10, 11). In the early stage of *Mycobacterium tuberculosis* infection, the host’s immune system initiates immune activation after recognizing the foreign pathogen-associated molecular patterns (PAMPs) (12–14), up-regulating the expression of pro-inflammatory cytokines such as IFNγ, TNFα, IL-17A, and IL-1β, MCP-1, and gradually activating the innate and adaptive immunity (15–17). The main cytokines IL-12 and IFN-γ are the reason for the activated Th1 cell response (18). However, if this immune activation effect is not properly controlled, the enormous production of cytokines (cytokine release storm, CRS) may cause serious damage to organs and tissues and even be fatal (19, 20). Current anti-tuberculosis drug combination therapy rarely considers the immunomodulatory activity of each drug, but the immune effect of these drugs for a long time is causing damage to organs and tissues, as well as the emergence of drug resistance. The different immune status of different TB patients, such as immunocompromised HIV co-infected patients, and the treatment strategy should also take into account the immune characteristics of anti-TB drugs themselves, which need to be further studied.

Some studies have found that different antibiotics exhibit different immunomodulatory activities and have different effects on treatment outcomes. Gross et al. demonstrated in a murine peritonitis model that bactericidal antibiotics produce more severe organ tissue damage caused by excessive immune activation than bacteriostatic antibiotics. The mechanism of action is that bactericidal antibiotics release more pathogen DNA, and the host recognizes and upregulates inflammatory immunity through TLR9, which ultimately reduces the survival of mice (21). In addition to being widely used in the treatment of tuberculosis, the immunosuppressive activity of rifampicin is also used in the treatment of heart transplantation and psoriasis (22–24). This inhibitory effect of rifampicin may be through affecting the IκB and MAPK signaling pathways (22). Pyrazinamide modulates the host immune response to Mtb infection in a PPAR- and NF-κB-dependent manner (25).

In this study, we evaluated the immunomodulatory activities of important anti-TB drugs using NF-kB and MAPK luciferase reporter systems and found that Macozinone (PBTZ169) exhibited potent innate immune activation. PBTZ169, an excellent derivative of benzothiazinones (BTZs), affects the synthesis of Mycobacterium cell wall by targeting the DprE1 enzyme (26–29). At present, PBTZ169 has entered clinical phase II (NCT03334734) and shows superior activity against Mtb and drug-resistant Mtb, with a good safety profile (30). PBTZ169 has the potential to be the core of the next-generation drug for drug-resistant TB treatment, but its effect on host immunity is still unknown.

We found that treatment of macrophages with PBTZ169 significantly promoted the expression of cytokines IL6, IL1β, TNFα, and type I interferons (IFNα and IFNβ), both in response to LPS stimulation and in response to infection with the PBTZ169-resistant strain, and this activation of innate immunity by PBTZ169 induced a decrease in intracellular Mtb colony-forming unit (CFU) counts. Further mechanistic study showed that PBTZ169 significantly promoted the phosphorylation of TAK1 in the NF-κB and MAPK signaling pathways. In summary, we have uncovered for the first time the role of PBTZ169 in the host immune activation and the maintenance of host immune homeostasis.

## RESULTS

### PBTZ169 promotes innate immune signaling pathways

**Fig 1:**
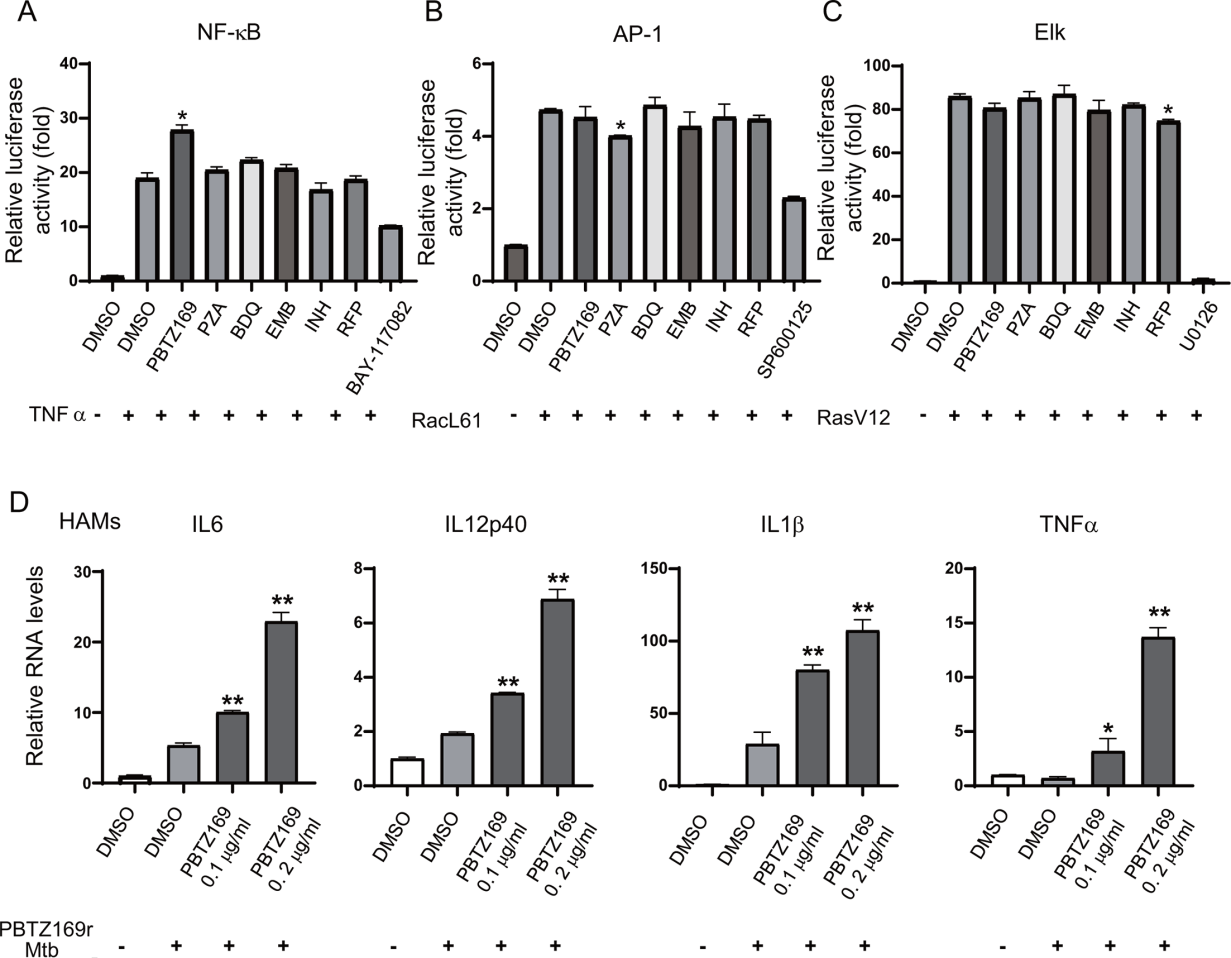
PBTZ169 promotes the activation of innate immune signaling pathways. (**A–C**) Luciferase assay of HEK293T cells transfected with plasmids encoding NF-kB(A), JNK(B), or ERK(C) luciferase reporters and treated with PBTZ169 (2 μg/mL), PZA (2 μg/mL), BDQ (2 μg/mL), EMB (2 μg/mL), INH (2 μg/mL), RFP (2 μg/mL), or DMSO (0.1%; control). The ERK pathway was activated by the co-expression of constitutively active RasV12. The JNK and p38 MAPK pathways were activated by co-expression of constitutively active RacL61. The NF-κB pathway was stimulated by TNF treatment. (**D**) Human alveolar macrophages (HAMs) were infected with PBTZ169r-Mtb at a MOI of 5 and treated with PBTZ169 (0.1 or 0.2 µg/mL) for 24 h. mRNA expression levels of IL-6, IL-1β, TNF-α, and IL-12p40 were quantified by qRT-PCR. (*n* = 3; means and SD; *, *P* < 0.05; **, *P* < 0.01).

To gain insight into the effect of TB therapeutic drugs on host innate immunity, we screened the innate immunomodulatory activity of clinically used or clinically investigated drugs using dual NF-kB and MAPK dual luciferase reporter systems. We found that PBTZ169 potentially upregulated innate immunity. In the NF-kB reporter system, the fluorescence intensity in the PBTZ169 treatment group was significantly higher than that in groups treated with equivalent concentrations of isoniazid (INH), rifampicin (RFP), pyrazinamide (PZA), ethambutol (EMB), or bedaquiline (BDQ) (Fig. 1A).

### PBTZ169 increases the expression of cytokines and type I interferon

Macrophages constitute the primary survival niche for *Mycobacterium tuberculosis* post-infection. As key components of the innate immune system, they are major cytokine sources and critical for activating other immune cells and adaptive immunity. To assess PBTZ169’s effect on cytokine production, we infected primary human alveolar macrophages (HAMs) with a clinically derived PBTZ169-resistant strain (PBTZ169r-Mtb) at MOI = 5, followed by 24 h of PBTZ169 treatment. RNA extraction and RT-qPCR revealed that PBTZ169 further elevated expression of IL-6, IL-12p40, IL-1β, and TNF-α (Fig. 1B).

**Fig 2:**
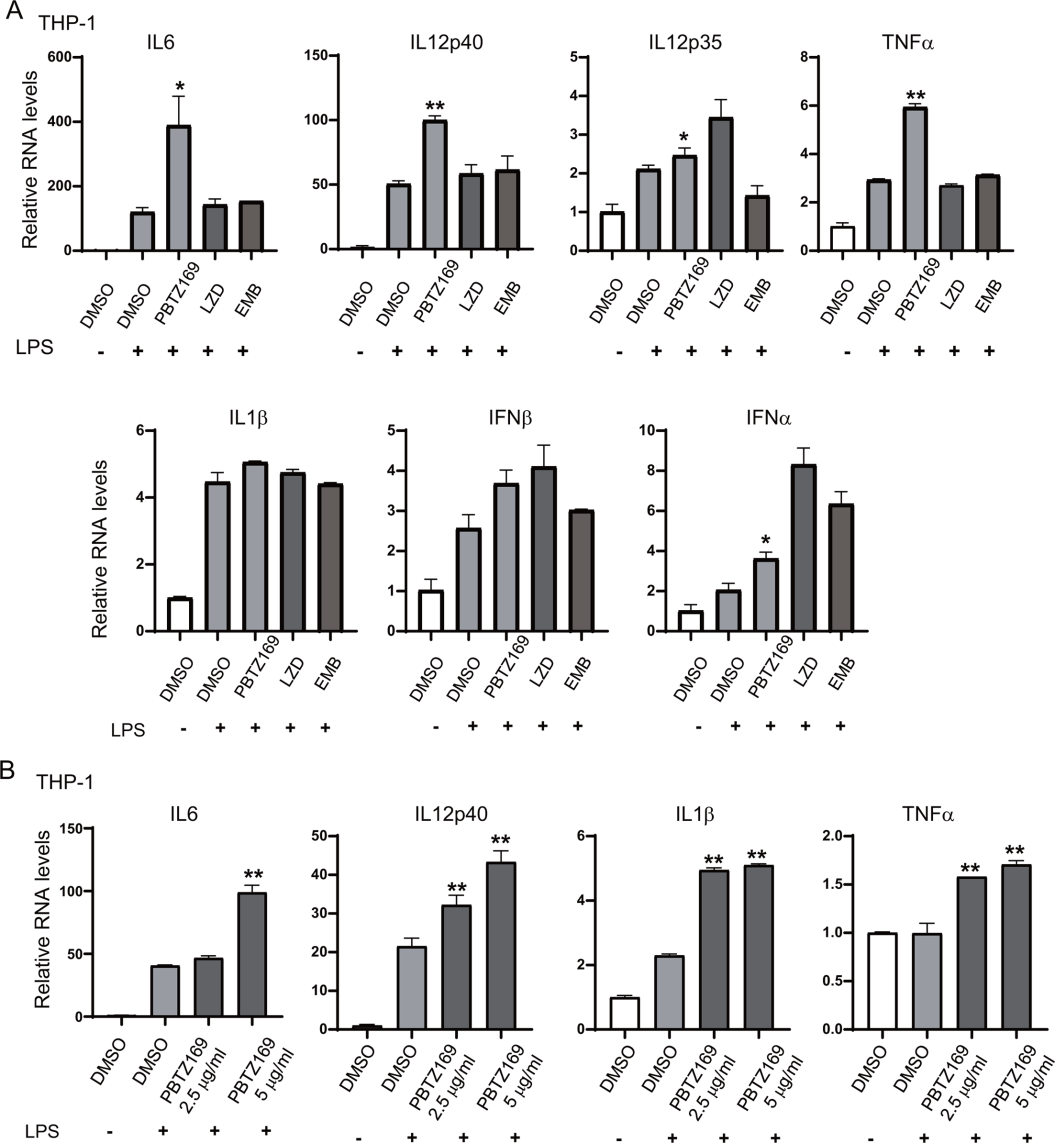
Under LPS stimulation, PBTZ169 improves the expression of cytokines and type I interferons. (**A**) PMA-induced THP-1 cells were pretreated with PBTZ169 (5 μg/mL), LZD (5 μg/mL), or EMB (5 μg/mL) for 8 h and then stimulated with lipopolysaccharide (LPS, 100 ng/mL) for an additional 12 h. The expression of cytokines IL6, IL12p40, IL12p35, TNFα, IL1β, and type I interferon IFNα and IFNβ was measured by RT-qPCR. (**B**) PMA-induced THP-1 cells were pretreated with PBTZ169(2.5 μg/mL) or PBTZ169(5 μg/mL) for 8 h and then treated with LPS(100 ng/mL) for another 12 h. mRNA levels of IL6, IL12p40, IL1β, and TNFα were analyzed. DMSO was used as a control. (*n* = 3; means and SD; *, *P* < 0.05; **, *P* < 0.01).

In parallel, we stimulated PMA-differentiated THP-1 cells with LPS to model infection, then treated them with PBTZ169. RT-qPCR analysis demonstrated significant upregulation of IL-1b, IL-6, IL-12p40, IL-12p35, TNF-α, IFN-α, and IFN-β by PBTZ169, unlike linezolid (LZD) or ethambutol (EMB) (Fig. 2A). Dose-response experiments confirmed this enhancement was concentration-dependent (Fig. 2B).

**Fig 3:**
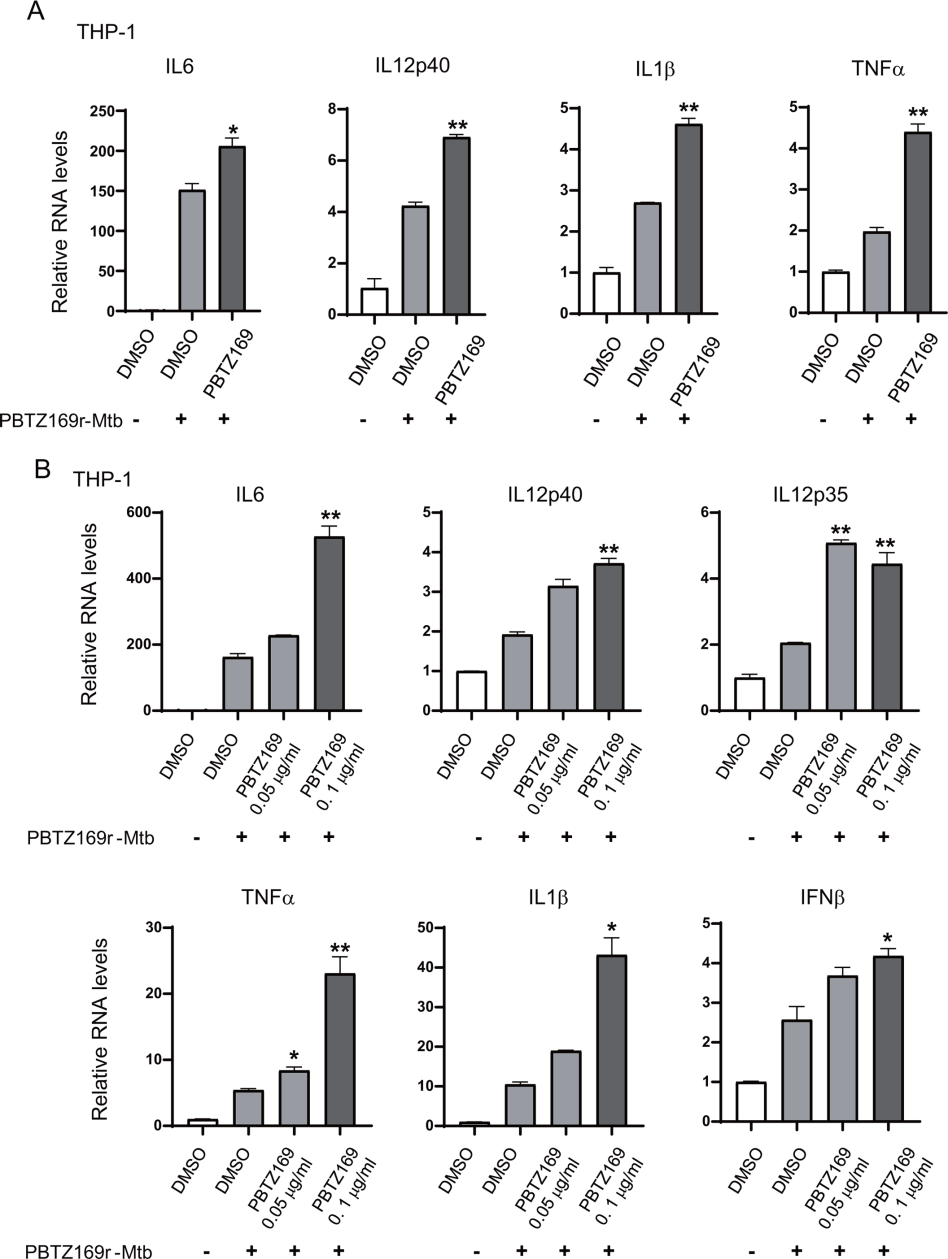
PBTZ169 increases the expression of cytokines and type I interferons after infection with PBTZ169r-Mtb. (**A**) Quantitative PCR analysis (RT-qPCR) of IL6, IL12p40, IL1β, and TNFα mRNA from THP-1 cells infected with PBTZ169-resistant Mtb (PBTZ169r-Mtb, MOI = 5) and treated with PBTZ169 (0.2 μg/mL) for 24 h. DMSO and uninfected groups were used as controls. (**B**) mRNA expression of IL6, IL12p40, IL12p35, TNFα, IL1β, and IFNβ from THP-1 cells infected with PBTZ169r-Mtb (MOI = 1) and treated with PBTZ169 (0.05 μg/mL) or PBTZ169 (0.1 μg/mL) for 5 days. DMSO and uninfected groups were used as controls. (*n* = 3; means and SD; *, *P* < 0.05; **, *P* < 0.01).

Furthermore, PMA-differentiated THP-1 cells infected with PBTZ169r-Mtb (MOI = 5) and treated with PBTZ169 for 24 h showed significantly increased RNA expression of IL-6, IL-12p40, IL-1β, and TNF-α (Fig. 3A).

To evaluate sustained innate immune activation, infected THP-1 macrophages (MOI = 1) were washed 4–6 h post-infection to remove non-phagocytosed bacteria and then cultured for 5 days with PBTZ169 or DMSO. PBTZ169 maintained cytokine-enhancing effects at 0.05 μg/mL and 0.1 μg/mL, dose-dependently upregulating IL-6, IL-12p40, IL-12p35, TNF-α, IL-1β, and IFN-β (Fig. 3B).

Collectively, these findings demonstrate that PBTZ169 robustly enhances innate immune activation during Mtb infection, increasing macrophage production of pro-inflammatory cytokines and type I interferons.

### The immunoenhancing activity of PBTZ169 potentiates intracellular bactericidal efficacy

**Fig 4:**
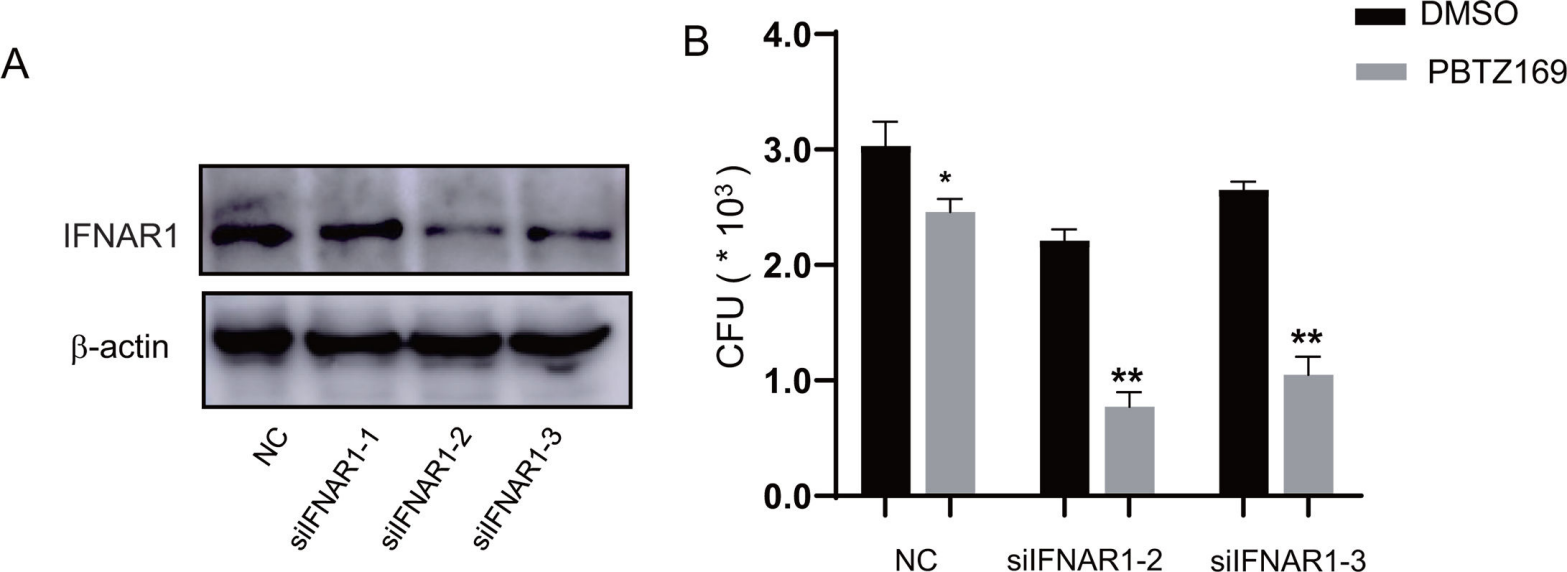
PBTZ169 inhibits the intracellular growth of PBTZ169r-Mtb. (**A**) J774A.1 cells were seeded into 24 wells and transfected with control siRNA (Negative Control, NC) or siRNA targeting IFNAR1 (50 nM) for 24 h, the efficiency of siRNA-based knockdown of IFNAR1 was determined by immunoblotting. (**B**) Intracellular colony-forming units (CFUs) assay in mouse IFNAR1-knockdown J774A.1 cells infected with PBTZ169r-Mtb (MOI = 5) for 4 h and treated with PBTZ169 (0.2 μg/mL) for another 24 h. An equal volume of DMSO was used as a control. (*n* = 3; means and SD; *, *P* < 0.05; **, *P* < 0.01).

Given that PBTZ169 greatly promotes the activation of host antimicrobial innate immunity, we sought to test the effect of this activity of PBTZ169 on intracellular bactericidal activity. We infected J774A.1 macrophages with PBTZr-169-Mtb and treated them with PBTZ169 for 48 h, then quantified bacterial colony-forming units (CFU) in the different treatment groups. PBTZ169 significantly reduced the intracellular survival of PBTZ169r-Mtb in a dose-dependent manner (Fig. 4B). To eliminate the interference of IFNAR1, we also knocked down the intracellular IFNAR1 with siRNA. Among them, the knockdown effects of siIFNAR1-2 and siIFNAR3 were relatively better (Fig. 4A). After knocking down IFNAR1, the ability of PBTZ169 to enhance the anti-Mtb activity of macrophages was further improved (Fig. 4B).

### RNA deep sequencing reveals that PBTZ169 has a significant activation effect on host innate immunity

**Fig 5:**
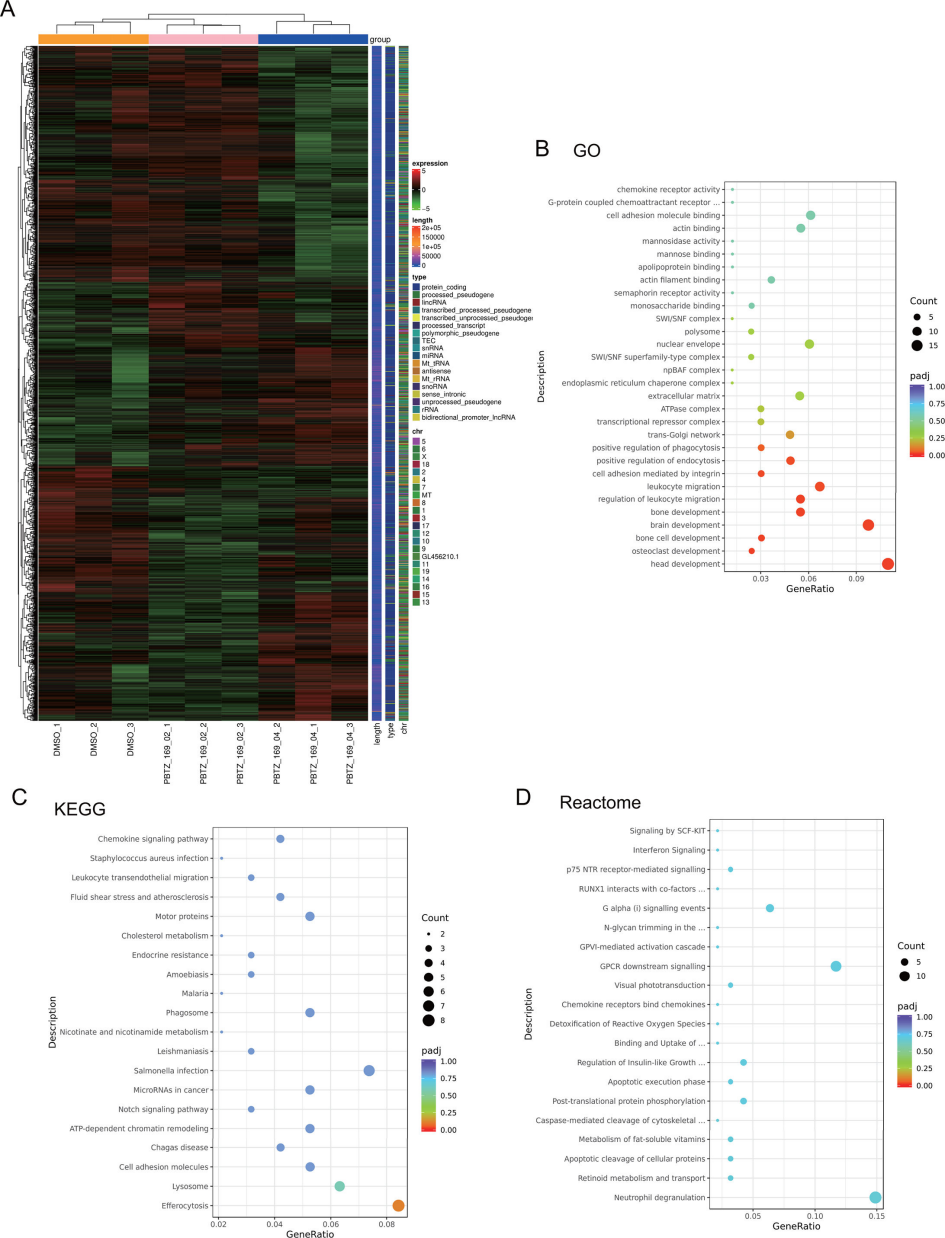
RNA deep sequencing reveals that PBTZ169 upregulates host innate immunity against Mtb. (**A**) The differentially expressed genes (DEGs) in J774A.1 cells infected with PBTZ169r-Mtb (MOI = 5) and treated with PBTZ169 (0.2 μg/mL), PBTZ169 (0.4 μg/mL), or DMSO were analyzed by RNA-seq. The heat map shows the differentially expressed genes in all groups. (**B–D**) The GO (**B**), KEGG (**C**), and Reactome (**D**) analyses of PBTZ169 0.2 μg group compared with the DMSO group. Shown are the signal pathways enriched by upregulated genes. Every group has three replicates.

To further investigate PBTZ169’s immunomodulatory mechanisms, we performed RNA sequencing on J774A.1. Cells infected with PBTZ169r-Mtb (4 h) were washed with PBS and then cultured with 0.2 μg/mL or 0.4 μg/mL PBTZ169 or DMSO control for 24 h (*n* = 3 replicates/group). We detected 453 and 564 differentially expressed genes (DEGs) for 0.2 μg/mL and 0.4 μg/mL PBTZ169 versus DMSO, respectively (Fig. 5A).

Gene Ontology (GO), Kyoto Encyclopedia of Genes and Genomes (KEGG), and Reactome enrichment analysis of DEGs revealed potent innate immune activation. For GO analysis, PBTZ169 0.2 μg/mL upregulated signaling pathways related to leukocyte migration, phagocytosis, and cytokine receptor activity (Fig. 5B). KEGG analysis showed enrichment in lysosome function, leukocyte transendothelial migration, chemokine signaling, phagosome formation, and efferocytosis (Fig. 5C). Reactome pathways significantly upregulated included neutrophil degranulation, chemokine receptors bind chemokines, and interferon signaling (Fig. 5D). Together, these data show that PBTZ169 activates multifaceted innate immune pathways during *Mtb* infection.

### PBTZ169 activates NF-kB and MAPK signaling pathways

**Fig 6:**
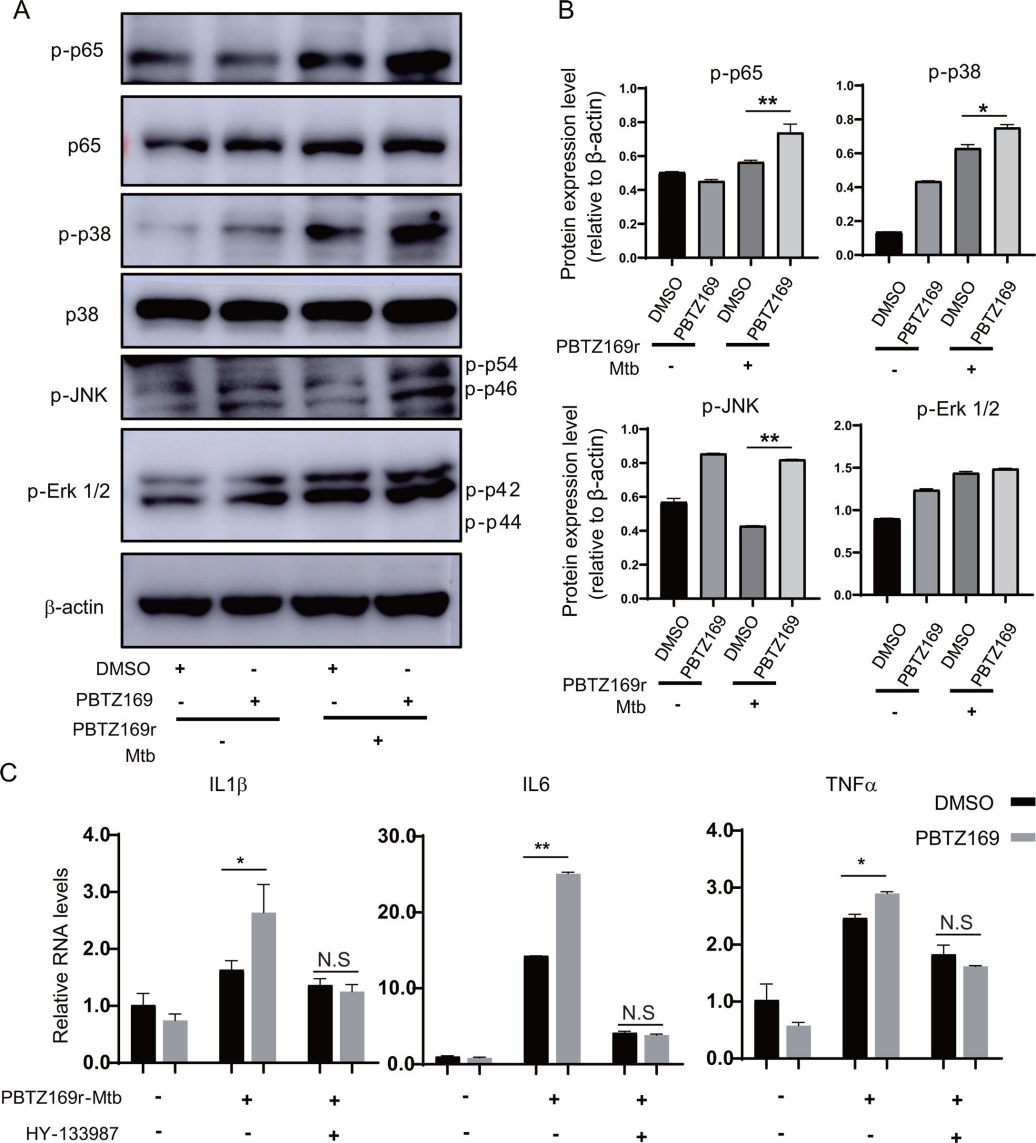
The upregulation of host innate immunity by PBTZ169 is dependent on the activation of NF-kB and MAPK. (**A**) Immunoblot detection of indicated proteins and their phosphorylation in PMA-induced THP-1 macrophages infected with or without PBTZ169r-Mtb (MOI = 5) and treated with PBTZ169 (0.2 μg/mL) or an equal volume of DMSO for 12 h. (**B**) Densitometry analysis of the western blots in Fig. 6A. (**C**) Quantitative PCR analysis of IL6, IL1β, and TNFα from J774A.1 cells. The cells were pretreated with or without AP-1/NF-kB activation inhibitor (HY-133987), infected with PBTZ169r-Mtb (MOI = 5), and treated with PBTZ169 (0.2 μg/mL) for 24 h. (*n* = 3; means and SD; *, *P* < 0.05; **, *P* < 0.01).

Having established that PBTZ169 can improve anti-Mtb innate immunity, we next sought to elucidate the mechanism driving this outcome. THP-1 cells were infected with PBTZ169r-Mtb (MOI = 5) for 4 h and continued to be treated with PBTZ169 (0.2 μg/mL) for another 12 h. Western blot analysis of key NF-kB (p65) and MAPK (p38, ERK, and JNK) pathway components revealed that PBTZ169 significantly increased phosphorylation of p65, p38, and JNK isoforms (p54/p46) compared with controls (Fig. 6A and B). Notably, this phosphorylation enhancement occurred even in uninfected cells.

To determine whether the observed effects of PBTZ169 require co-activation of NF-kB and MAPK pathway, we assessed cytokine production following simultaneous administration of PBTZ169 with a dual inhibitor of NF-kB and AP-1, ethyl 4-[(3-methyl-2,5-dioxo(3-pyrrolinyl))amino]−2-(trifluoromethyl)pyrimidine-5-carboxylate) (31). As expected, PBTZ169 significantly increased IL-1β, IL6, and TNFα in the absence of inhibitors. However, NF-κB and AP-1 inhibitors co-treatment abolished this upregulation, demonstrating that PBTZ169’s immunostimulatory activity is NF-κB/MAPK-dependent (Fig. 6C).

### The immune-activating effect of PBTZ169 is dependent on TAK1

**Fig 7:**
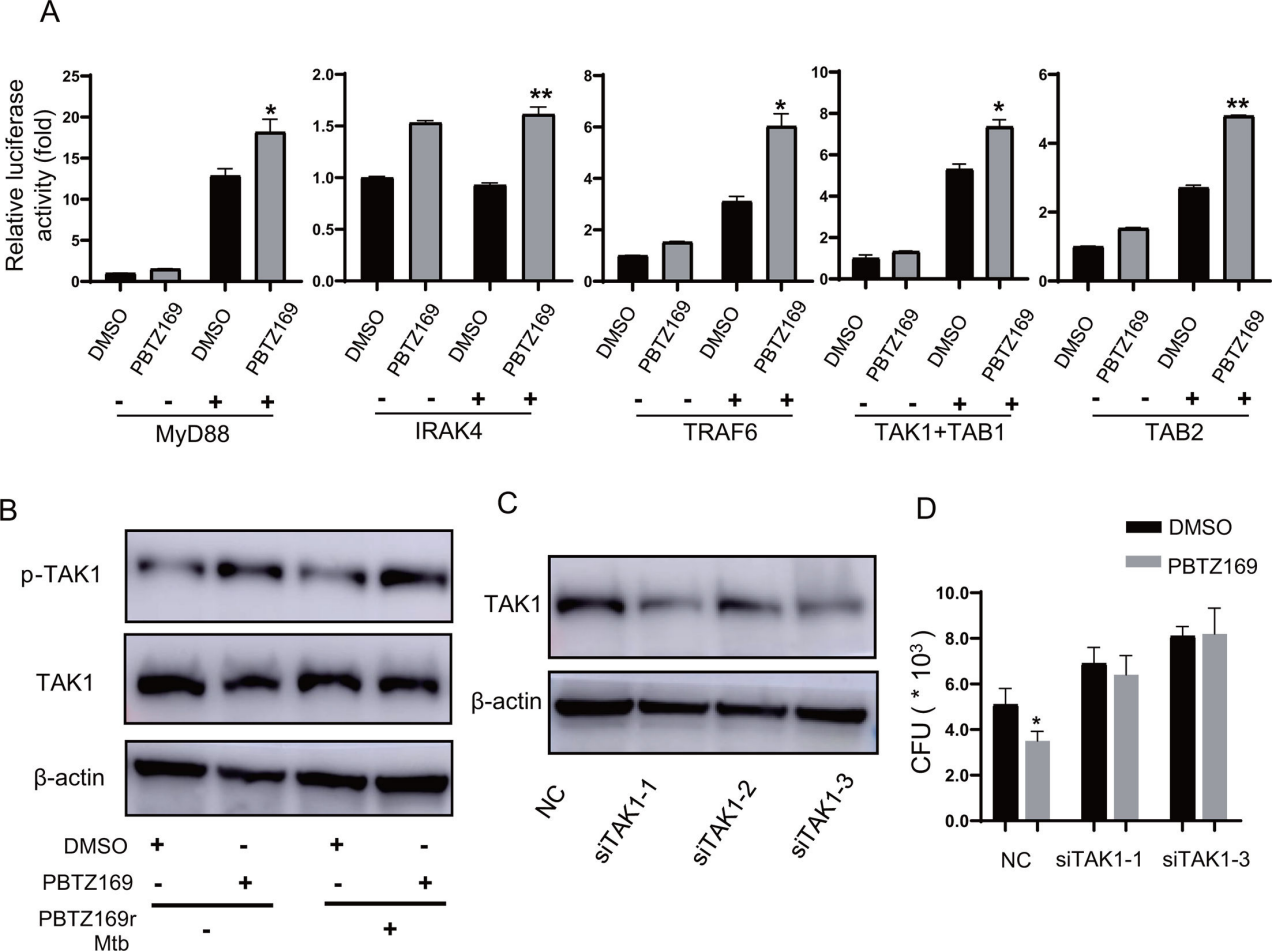
PBTZ169 upregulates antibacterial immunity by activating TAK1. (**A**) The dual fluorescence activation level of the NF-kB by PBTZ169, the NF-kB dual fluorescence system, is activated by MyD88, IRAK4, TRAF6, TAK1, or TAB2. (**B**) Immunoblot detection of TAK1 and its phosphorylation in PMA-induced THP-1 macrophages infected with or without PBTZ169r-Mtb (MOI = 5) and treated with PBTZ169 (0.2 μg/mL) or an equal volume of DMSO for 12 h. (**C**) TAK1 was knocked down by siRNA, and the knockdown efficiency was determined by immunoblotting assay. (**D**) J774A.1 cells were transfected with siRNA control (negative control, NC) or siRNA targeting TAK1 for 24 h, infected with PBTZ169r-Mtb (MOI = 5) for 4 h, and treated with PBTZ169(0.2μg/mL) for another 24 h. Cells were prepared for the CFU assay. (*n* = 3; means and SD; *, *P* < 0.05; **, *P* < 0.01).

Next, we proceeded to decode the mechanisms whereby PBTZ169 promotes MAPK and NF-kB pathways and thereby enhances intracellular bactericidal efficacy, and we probed its interaction with key components in the Toll-like receptors signaling cascade. The luciferase assay showed that PBTZ169 led to a significant increase in NF-kB luciferase activation induced by MyD88, IRAK4, TRAF6, TAK1, or TAB2 (Fig. 7A). Given TAK1’s role as a convergence point for NF-κB and MAPK pathways, we assessed its activation status. Strikingly, PBTZ169 significantly increased TAK1 phosphorylation (Thr184/187) in PBTZ169r-Mtb-infected THP-1 cells (Fig. 7B).

To determine whether the immune activation effect of PBTZ169 depends on the activation of TAK1, we knocked down TAK1 in J774A.1 cells and observed that the phenomenon of PBTZ169 enhancing intracellular bactericidal activity markedly decreased. In conclusion, our data demonstrate that PBTZ169 activates the host’s antimicrobial innate immunity by promoting TAK1 phosphorylation.

## DISCUSSION

Macozinone (MCZ, PBTZ169) is a new drug for the treatment of TB, currently in clinical phase II study, that inhibits mycobacterial cell wall synthesis by covalently targeting the crucial DprE1 enzyme (32, 33). In preclinical and clinical studies, PBTZ169 has shown superior activity against sensitive tuberculosis, multidrug-resistant tuberculosis, and extensively drug-resistant tuberculosis and is the most prominent candidate drug in the benzothiazinone class (26–28, 30, 32, 34–36). In addition to its activity against tuberculosis, the current study of PBTZ169 has potentially high therapeutic activity against both *Mycobacterium fortuitum* (37) and *Pseudomonas aeruginosa* (38). In the TB hypoxic granuloma model, PBTZ169 was more potent than rifampicin in reducing the CFU counts and decreasing the bacterial burden across mouse models, manifesting as early as four weeks into the treatment regimen (30). However, the effect of PBTZ169 on the regulation of host innate immune activity and the production of cytokines and interferons has not been reported. We found for the first time that PBTZ169 upregulated the expression of cytokines IL6, IL1β, IL12, TNFα, IFNα, and IFNβ in both LPS-simulated and Mtb infection conditions.

Host immune homeostasis deserves increasing attention in TB treatment. It is estimated that 30%–50% of TB patients worldwide develop various forms of post-TB lung disease after treatment, potentially related to the excessive production of cytokines and chemokines during treatment. Tuberculosis causes long-term chronic inflammation and fibrosis, which may lead to gene mutations and other cellular changes (39–41). Factors such as the involvement of parenchymal lung tissue by tuberculosis, lymphoproliferative processes, morphological variations of blood vessels, and the production of immune system mediators, such as interleukins, contribute to the hypothesis of a link between tuberculosis and lung cancer (19, 42–44). Different drugs exert distinct immunomodulatory effects on the host. In this study, we found that PBTZ169 upregulated the host innate immunity by activating NF-kB and MAPK, which may have important implications for the reconsideration of tuberculosis treatment. Therefore, drug selection may need to be tailored based on immunomodulatory activity, considering individual patient differences and treatment stage, to achieve optimal therapeutic outcomes. Of course, this concept remains in its early stages and requires validation through additional clinical trials and further research grounded in patient data.

Cytokines and chemokines are important mediators of various immune responses. If they are not well controlled, they may cause permanent damage to the patient’s body, which is not conducive to the treatment of the disease. Joshi et al. found that the levels of TNF-α, IL-6, and IL-10 were correlated with the severity of TB (19, 45). Di et al. reported significantly elevated serum levels of TNF-α, IL-6, IFN-γ, sIL-2R, and CRP in COPD patients with TB compared with other groups, which were correlated with greater disease severity and a poorer prognosis (46). Kumar et al. found that PTB individuals with slower culture conversion displayed significantly elevated levels of CCL1, CCL3, CXCL1, and CXCL9 at the time of TB diagnosis and prior to anti-tuberculous chemotherapy (47). The immune activation of PBTZ169 may further aggravate the inflammatory injury caused by Mtb infection. Therefore, we hypothesized that its antibacterial and pro-inflammatory activity may be more suitable for the early stage of Mtb infection and the establishment of defensive immunity, but the risk of CRS should be paid attention to. Our study will provide a theoretical basis for the appropriate clinical use of PBTZ169 and other antituberculosis drugs.

## MATERIALS AND METHODS

### Cell lines and mycobacterial strains

The HEK293T cells (ATCC CRL-3216, RRID: CVCL_0063) and J774A.1 cells (ATCC TIB-67, RRID: CVCL_0358) were cultured in Dulbecco’s-modified Eagle’s medium (Gibco, USA) supplemented with 10% fetal bovine serum (Gibco, USA). The THP-1 cells (ATCC TIB-202, RRID: CVCL_0006) were maintained in RPMI-1640 (Gibco, USA) with 10% FBS and differentiated into adherent macrophage-like cells with 10 ng/mL phorbol 12-myristate 13-acetate (PMA) overnight, then the cells were washed once with PBS and cultured in fresh RPMI-1640 medium. The primary human alveolar macrophages were purchased from MeisenCTCC (Hangzhou, China). All cells were cultured at 37°C in 5% CO_2_.

*Mycobacterium tuberculosis* H37Rv (ATCC 27294) and the Clinical PBTZ169-resistant strain (PBTZ169r-Mtb) for this study were obtained from the National Clinical Laboratory on Tuberculosis in Beijing Chest Hospital. H37Rv and PBTZ169r-Mtb were cultured in Middlebrook 7H9 broth with 0.2% (vol/vol) glycerol, 0.05% Tween-80, and 10% (vol/vol) oleic acid-albumin-dextrose-catalase (OADC).

### Drugs, plasmids, and antibodies

INH, RFP, PZA, EMB, and LZD were purchased from Sigma-Aldrich (Missouri, USA). BDQ and PBTZ169 were purchased from Biochempartner (Shanghai, China). AP-1/NF-κB activation inhibitor 1 (HY-133987) was purchased from MedChemExpress (New Jersey, USA).

NF-kB and MAPK/JNK activation luciferase reporter plasmids (pRL-TK, Gal4-Elk, Gal4-luc, pFA-cJun, RacL61, pNF-kB-luc, and RasV12) were kindly provided by Cuihua Liu (Institute of Microbiology, Chinese Academy of Sciences). The MyD88, IRAK4, TRAF6, TAK1, and TAB2 overexpression plasmids were constructed by our laboratory and sequenced by RuiBiotech (Beijing, China).

Antibodies against p38 MAPK (#8690), phospho-p38 MAPK (Thr180/Tyr182, #4511), phospho-NF-κB p65 (Ser536,#3033), NF-κB p65 (#8242), p44/42 MAPK (Erk1/2, #4695), Phospho-p44/42 MAPK (Erk1/2) (Thr202/Tyr204,#9101), SAPK/JNK (#9252), phospho-SAPK/JNK (Thr183/Tyr185, #4668), and phospho-TAK1 (Thr184/187, #4508) were purchased from Cell Signaling Technology (Beverly, USA). Antibodies against β-actin (#GB11001) and GAPDH (#GB11002) were purchased from Servicebio (Wuhan, China). Goat Anti-Rabbit IgG-HRP (#P03S02) was purchased from Gene Protein Link (Beijing, China).

### Luciferase reporter assays

The NF-kB and MAPK dual luciferase reporter assays were performed as previously described (48–50). Briefly, pNF-kB-luc and pRL-TK plasmids were transfected into HEK293T cells cultured in 24-well plates at 70%–80% confluence. After 6 h, TNFα (20 ng/mL) was added to the fresh culture medium together with the test drug, and the cells were cultured for another 24 h, and the supernatant was removed. The cells were lysed, followed by dual-luciferase activity assays using a dual-luciferase reporter assay kit (Promega) according to the manufacturer’s instructions. Luciferase activity was normalized to that of Renilla luciferase activity. To measure the JNK pathway, Gal4-luc, pFA-cJun, RacL61, and pRL-TK were cotransfected into HEK293T cells. To measure the ERK pathway, Gal4-luc (0.3 μg), Gal4-Elk, pRL-TK, and RasV12 were cotransfected into HEK293T cells.

### RNA sequencing

J774A.1 cells infected with PBTZ169r-Mtb (4 h) were washed with PBS three times, then cultured with 0.2 μg/mL or 0.4 μg/mL PBTZ169 or DMSO for 24 h (*n* = 3 replicates/group). Following RNA extraction, sample quality control, library preparation, and different libraries were pooled based on the effective concentration and targeted data amount, then subjected to Illumina sequencing (Novogene, Beijing, China). Differentially expressed genes (DEGs) were identified (*P* value <=0.05). GO, Kyoto Encyclopedia of Genes and Genomes (KEGG), and Reactome were used for functional enrichment analysis of DEGs.

### RT-qPCR

Total RNA from cells was isolated by TRIzol reagent (Life Technologies) according to the manufacturer’s instructions, and the purity and quantity of RNA were assessed using DeNovix. Complementary DNA was prepared by the cDNA Synthesis kit (Yeasen, China). The indicated genes were amplified by SYBR Green Master Mix (Yeasen, China) using the ABI StepOnePlus Real-Time PCR System. GAPDH was used as a reference gene for internal standardization. For quantification, the 2^-ΔΔCt^ method was used to calculate the relative RNA levels against GAPDH.

### Western blotting

After removal of the culture supernatant, cells were washed once with precooled PBS and lysed for 20 min on ice with RIPA lysis buffer (R0010, Solarbio, China) supplemented with phosphatase inhibitors (P1051, Beyotime, China) and protease inhibitors (P1010, Beyotime, China). Then, after adding the loading buffer, the extracts were incubated in a heat block at 100°C for 10 min. Different protein samples were loaded onto the gel and subjected to SDS-PAGE, transferred onto PVDF membranes, and immunoblotted with primary antibodies listed above. Secondary antibodies conjugated to horseradish peroxidase were added, and luminescence was quantified using BeyoECL reagent (P0018, Beyotime, China).

### Intracellular Mtb replication

The Mtb standard strain, H37Rv, and the PBTZ169-resistant strain were grown to logarithmic growth phase in Middlebrook 7H9 medium with 0.05% Tween-80 (Sigma-Aldrich, P1754) and 10% OADC. PMA-differentiated THP-1 or J774A.1 cells were seeded with 1 × 10^6^ cells per well and infected with Mtb. After 4–6 h of infection, non-phagocytosed bacteria were removed, and the cells were washed three times with PBS, and the cells were then cultured in fresh medium with the assay drug for the indicated times. The cell medium was removed and washed three times with PBS, and the cells were lysed with SDS cell lysis buffer. Original lysates or 10-fold serial dilutions of cultures were spread onto Middlebrook 7H10 agar supplemented with 10% (vol/vol) OADC and 0.2% (vol/vol) glycerol for 3–4  weeks. Differences in intracellular growth of Mtb were assessed by counting colony-forming unit (CFU).

### Statistical analysis

All data were presented as the mean ± standard deviation. Statistical analysis was performed using GraphPad Prism 8 software (GraphPad Software, Inc.). One-way or two-way ANOVA with Dunnett’s correction was used for multiple comparisons. Student’s *t*-test was used for two-group comparisons. Statistical significance is indicated as follows: *P < 0.05, **P < 0.01.

## Data Availability

The data sets generated during and/or analyzed during the current study are available from the corresponding author on reasonable request.

## References

[1] Prabowo SA, Gröschel MI, Schmidt EDL, Skrahina A, Mihaescu T, Hastürk S, Mitrofanov R, Pimkina E, Visontai I, de Jong B, Stanford JL, Cardona P-J, Kaufmann SHE, van der Werf TS. 2013. Targeting multidrug-resistant tuberculosis (MDR-TB) by therapeutic vaccines. Med Microbiol Immunol 202:95–104. doi:10.1007/s00430-012-0278-6 23143437

[2] Marimani M, Ahmad A, Duse A. 2018. The role of epigenetics, bacterial and host factors in progression of Mycobacterium tuberculosis infection. Tuberculosis (Edinb) 113:200–214. doi:10.1016/j.tube.2018.10.009 30514504

[3] Schwarzlose-Schwarck S, Reinwald M, Bauer T, Hentschel F, Kiderlen T, Zapf D, Herbst V, Lüth S, Krieger D, Dammermann W. 2025. Evaluation of three novel antigens and costimulatory agents for improvement of M. tuberculosis specific interferon gamma release assays. BMC Infect Dis 25:188. doi:10.1186/s12879-025-10577-3 39920589 PMC11806546

[4] Wynn EA, Dide-Agossou C, Al Mubarak R, Rossmassler K, Ektnitphong V, Bauman AA, Massoudi LM, Voskuil MI, Robertson GT, Moore CM, Walter ND. 2025. Emergence of antibiotic-specific Mycobacterium tuberculosis phenotypes during prolonged treatment of mice. Antimicrob Agents Chemother. doi:10.1128/aac.01310-24:e0131024 PMC11823617 39818957

[5] Ukoaka BM, Daniel FM, Wagwula PM, Ahmed MM, Udam NG, Okesanya OJ, Babalola A, Wali TA, Afolabi S, Udoh RA, Peter IG, Maaji LA. 2024. Prevalence, clinical characteristics, and treatment outcomes of childhood tuberculosis in Nigeria: a systematic review and meta-analysis. BMC Infect Dis 24:1447. doi:10.1186/s12879-024-10321-3 39702126 PMC11656894

[6] Suárez I, Fünger SM, Kröger S, Rademacher J, Fätkenheuer G, Rybniker J. 2019. The diagnosis and treatment of tuberculosis. Dtsch Arztebl Int 116:729–735. doi:10.3238/arztebl.2019.0729 31755407

[7] Tiberi S, du Plessis N, Walzl G, Vjecha MJ, Rao M, Ntoumi F, Mfinanga S, Kapata N, Mwaba P, McHugh TD, Ippolito G, Migliori GB, Maeurer MJ, Zumla A. 2018. Tuberculosis: progress and advances in development of new drugs, treatment regimens, and host-directed therapies. Lancet Infect Dis 18:e183–e198. doi:10.1016/S1473-3099(18)30110-5 29580819

[8] Sweeney S, Laurence YV, Berry C, Singh MP, Dodd M, Fielding K, Kazounis E, Moodliar R, Solodovnikova V, Tigay Z, Liverko I, Parpieva N, Butabekov I, Usmanova R, Rassool M, Motta I, Nyangweso GM, Jolivet P, Abdrasuliev T, Moe S, Aw PS, Samieva N, Nyang’wa B-T. 2025. 24-week, all-oral regimens for pulmonary rifampicin-resistant tuberculosis in TB-PRACTECAL trial sites: an economic evaluation. Lancet Glob Health 13:e355–e363. doi:10.1016/S2214-109X(24)00467-4 39890235

[9] Nyang’wa B-T, Berry C, Kazounis E, Motta I, Parpieva N, Tigay Z, Moodliar R, Dodd M, Solodovnikova V, Liverko I, Rajaram S, Rassool M, McHugh T, Spigelman M, Moore DA, Ritmeijer K, du Cros P, Fielding K, TB-PRACTECAL team. 2024. Short oral regimens for pulmonary rifampicin-resistant tuberculosis (TB-PRACTECAL): an open-label, randomised, controlled, phase 2B-3, multi-arm, multicentre, non-inferiority trial. Lancet Respir Med 12:117–128. doi:10.1016/S2213-2600(23)00389-2 37980911

[10] York AG, Skadow MH, Oh J, Qu R, Zhou QD, Hsieh W-Y, Mowel WK, Brewer JR, Kaffe E, Williams KJ, Kluger Y, Smale ST, Crawford JM, Bensinger SJ, Flavell RA. 2024. IL-10 constrains sphingolipid metabolism to limit inflammation. Nature 627:628–635. doi:10.1038/s41586-024-07098-5 38383790 PMC10954550

[11] Afonina IS, Zhong Z, Karin M, Beyaert R. 2017. Limiting inflammation-the negative regulation of NF-κB and the NLRP3 inflammasome. Nat Immunol 18:861–869. doi:10.1038/ni.3772 28722711

[12] Swanson KV, Deng M, Ting JP-Y. 2019. The NLRP3 inflammasome: molecular activation and regulation to therapeutics. Nat Rev Immunol 19:477–489. doi:10.1038/s41577-019-0165-0 31036962 PMC7807242

[13] Ibrahim A, Saleem N, Naseer F, Ahmed S, Munawar N, Nawaz R. 2024. From cytokines to chemokines: understanding inflammatory signaling in bacterial meningitis. Mol Immunol 173:117–126. doi:10.1016/j.molimm.2024.07.004 39116800

[14] Killick KE, Ní Cheallaigh C, O’Farrelly C, Hokamp K, MacHugh DE, Harris J. 2013. Receptor-mediated recognition of mycobacterial pathogens. Cell Microbiol 15:1484–1495. doi:10.1111/cmi.12161 23795683

[15] Kumar NP, Moideen K, Banurekha VV, Nair D, Babu S. 2019. Plasma proinflammatory cytokines are markers of disease severity and bacterial burden in pulmonary tuberculosis. Open Forum Infect Dis 6:ofz257. doi:10.1093/ofid/ofz257 31281858 PMC6602384

[16] Lee JS, Song CH, Lim JH, Lee KS, Kim HJ, Park JK, Paik TH, Jung SS, Jo EK. 2003. Monocyte chemotactic protein-1 production in patients with active pulmonary tuberculosis and tuberculous pleurisy. Inflamm Res 52:297–304. doi:10.1007/s00011-003-1176-6 12861395

[17] Ruhwald M, Bjerregaard-Andersen M, Rabna P, Eugen-Olsen J, Ravn P. 2009. IP-10, MCP-1, MCP-2, MCP-3, and IL-1RA hold promise as biomarkers for infection with M. tuberculosis in a whole blood based T-cell assay. BMC Res Notes 2:19. doi:10.1186/1756-0500-2-19 19193208 PMC2660909

[18] Lyadova IV, Panteleev AV. 2015. Th1 and Th17 cells in tuberculosis: protection, pathology, and biomarkers. Mediators Inflamm 2015:854507. doi:10.1155/2015/854507 26640327 PMC4657112

[19] Boni FG, Hamdi I, Koundi LM, Shrestha K, Xie J. 2022. Cytokine storm in tuberculosis and IL-6 involvement. Infect Genet Evol 97:105166. doi:10.1016/j.meegid.2021.105166 34861432

[20] Tang S, Cui H, Yao L, Hao X, Shen Y, Fan L, Sun H, Zhang Z, Huang JA. 2013. Increased cytokines response in patients with tuberculosis complicated with chronic obstructive pulmonary disease. PLoS One 8:e62385. doi:10.1371/journal.pone.0062385 23626814 PMC3633855

[21] Gross JL, Basu R, Bradfield CJ, Sun J, John SP, Das S, Dekker JP, Weiss DS, Fraser IDC. 2024. Bactericidal antibiotic treatment induces damaging inflammation via TLR9 sensing of bacterial DNA. Nat Commun 15:10359. doi:10.1038/s41467-024-54497-3 39609397 PMC11605096

[22] Bi W, Zhu L, Wang C, Liang Y, Liu J, Shi Q, Tao E. 2011. Rifampicin inhibits microglial inflammation and improves neuron survival against inflammation. Brain Res 1395:12–20. doi:10.1016/j.brainres.2011.04.019 21555117

[23] Tsankov N, Grozdev I, Kkzandjieva J. 2006. Old drug – new indication. Rifampicin in psoriasis. J Dermatolog Treat 17:18–23. doi:10.1080/09546630500375643 16467019

[24] Tsai YC, Tsai TF. 2019. A review of antibiotics and psoriasis: induction, exacerbation, and amelioration. Expert Rev Clin Pharmacol 12:981–989. doi:10.1080/17512433.2019.1665027 31498683

[25] Manca C, Koo MS, Peixoto B, Fallows D, Kaplan G, Subbian S. 2013. Host targeted activity of pyrazinamide in Mycobacterium tuberculosis infection. PLoS One 8:e74082. doi:10.1371/journal.pone.0074082 24015316 PMC3755974

[26] Makarov V, Mikušová K. 2020. Development of macozinone for TB treatment: an update. Appl Sci (Basel) 10:2269. doi:10.3390/app10072269

[27] Makarov V, Manina G, Mikusova K, Möllmann U, Ryabova O, Saint-Joanis B, Dhar N, Pasca MR, Buroni S, Lucarelli AP, et al.. 2009. Benzothiazinones kill Mycobacterium tuberculosis by blocking arabinan synthesis. Science 324:801–804. doi:10.1126/science.1171583 19299584 PMC3128490

[28] Neres J, Pojer F, Molteni E, Chiarelli LR, Dhar N, Boy-Röttger S, Buroni S, Fullam E, Degiacomi G, Lucarelli AP, Read RJ, Zanoni G, Edmondson DE, De Rossi E, Pasca MR, McKinney JD, Dyson PJ, Riccardi G, Mattevi A, Cole ST, Binda C. 2012. Structural basis for benzothiazinone-mediated killing of Mycobacterium tuberculosis. Sci Transl Med 4:150ra121. doi:10.1126/scitranslmed.3004395 PMC3659392 22956199

[29] Batt SM, Jabeen T, Bhowruth V, Quill L, Lund PA, Eggeling L, Alderwick LJ, Fütterer K, Besra GS. 2012. Structural basis of inhibition of Mycobacterium tuberculosis DprE1 by benzothiazinone inhibitors. Proc Natl Acad Sci USA 109:11354–11359. doi:10.1073/pnas.1205735109 22733761 PMC3396498

[30] Nikonenko B, Logunova N, Egorova A, Kapina M, Sterzhanova N, Bocharova I, Kondratieva E, Riabova O, Semyonova L, Makarov V, Dedicated to the 10th anniversary of the iM4TB Foundation. 2024. Efficacy of macozinone in mice with genetically diverse susceptibility to Mycobacterium tuberculosis infection. Microbes Infect 26:105376. doi:10.1016/j.micinf.2024.105376 38852904

[31] Palanki MS, Erdman PE, Manning AM, Ow A, Ransone LJ, Spooner C, Suto C, Suto M. 2000. Novel inhibitors of AP-1 and NF-κB mediated gene expression: structure–activity relationship studies of ethyl 4-[(3-Methyl-2,5-dioxo(3-pyrrolinyl))amino]-2-(trifluoromethyl)pyrimidine-5-carboxylate. Bioorg Med Chem Lett 10:1645–1648. doi:10.1016/s0960-894x(00)00312-7 10937715

[32] Lupien A, Vocat A, Foo CS-Y, Blattes E, Gillon J-Y, Makarov V, Cole ST. 2018. Optimized background regimen for treatment of active tuberculosis with the next-generation benzothiazinone macozinone (PBTZ169). Antimicrob Agents Chemother 62:e00840-18. doi:10.1128/AAC.00840-18 30126954 PMC6201121

[33] Lechartier B, Cole ST. 2015. Mode of action of clofazimine and combination therapy with benzothiazinones against Mycobacterium tuberculosis. Antimicrob Agents Chemother 59:4457–4463. doi:10.1128/AAC.00395-15 25987624 PMC4505229

[34] Makarov V, Lechartier B, Zhang M, Neres J, van der Sar AM, Raadsen SA, Hartkoorn RC, Ryabova OB, Vocat A, Decosterd LA, Widmer N, Buclin T, Bitter W, Andries K, Pojer F, Dyson PJ, Cole ST. 2014. Towards a new combination therapy for tuberculosis with next generation benzothiazinones. EMBO Mol Med 6:372–383. doi:10.1002/emmm.201303575 24500695 PMC3958311

[35] Guo S, Fu L, Wang B, Chen X, Zhao J, Liu M, Lu Y. 2020. In vitro and in vivo antimicrobial activities of a novel piperazine-containing benzothiazinones candidate TZY-5-84 against Mycobacterium tuberculosis. Biomed Pharmacother 131:110777. doi:10.1016/j.biopha.2020.110777 33152936

[36] Lv K, Tao Z, Liu Q, Yang L, Wang B, Wu S, Wang A, Huang M, Liu M, Lu Y. 2018. Design, synthesis and antitubercular evaluation of benzothiazinones containing a piperidine moiety. Eur J Med Chem 151:1–8. doi:10.1016/j.ejmech.2018.03.060 29601990

[37] Roberto Tavolari Jortieke C, Rocha Joaquim A, Fumagalli F. 2024. Advances in antibacterial agents for Mycobacterium fortuitum. RSC Med Chem 16:37–49. doi:10.1039/d4md00508b 39493226 PMC11528911

[38] Joshi T, Vijayakumar S, Ghosh S, Mathpal S, Ramaiah S, Anbarasu A. 2024. Identifying novel therapeutics for the resistant mutant “F533L” in PBP3 of Pseudomonas aeruginosa using ML techniques. ACS Omega 9:28046–28060. doi:10.1021/acsomega.4c00929 38973840 PMC11223260

[39] Woldesemayat EM, Vera JH, Tanner C, Tamiso A, Assefa A, Woldesenbet YM. 2025. Lung function of tuberculosis patients after completion of treatment in Sidama, South Ethiopia. Front Med (Lausanne) 12:1451861. doi:10.3389/fmed.2025.1451861 40171505 PMC11958218

[40] Thoker ZA, Madan K, Mittal S, Tiwari P, Shah TH, Mohan A, Hadda V, Guleria R. 2023. Clinical profile and quality of life of patients with post-pulmonary tuberculosis sequelae presenting to a Tertiary care hospital. Cureus 15:e36354. doi:10.7759/cureus.36354 37082491 PMC10112386

[41] Singh S, Allwood BW, Chiyaka TL, Kleyhans L, Naidoo CC, Moodley S, Theron G, Segal LN. 2022. Immunologic and imaging signatures in post tuberculosis lung disease. Tuberculosis (Edinb) 136:102244. doi:10.1016/j.tube.2022.102244 36007338 PMC10061373

[42] Engels EA, Shen M, Chapman RS, Pfeiffer RM, Yu YY, He X, Lan Q. 2009. Tuberculosis and subsequent risk of lung cancer in Xuanwei, China. Int J Cancer 124:1183–1187. doi:10.1002/ijc.24042 19058197 PMC2610239

[43] Fried LE, Arbiser JL. 2009. Honokiol, a multifunctional antiangiogenic and antitumor agent. Antioxid Redox Signal 11:1139–1148. doi:10.1089/ars.2009.2440 19203212 PMC2842137

[44] Liang HY, Li XL, Yu XS, Guan P, Yin ZH, He QC, Zhou BS. 2009. Facts and fiction of the relationship between preexisting tuberculosis and lung cancer risk: a systematic review. Int J Cancer 125:2936–2944. doi:10.1002/ijc.24636 19521963

[45] Joshi L, Ponnana M, Sivangala R, Chelluri LK, Nallari P, Penmetsa S, Valluri V, Gaddam S. 2025. Correction: evaluation of TNF-α, IL-10 and IL-6 cytokine production and their correlation with genotype variants amongst tuberculosis patients and their household contacts. PLoS One 20:e0318949. doi:10.1371/journal.pone.0318949 39903750 PMC11793787

[46] Di Y, Yang F, Che C, Xu S, Qi Y. 2025. Correlation between serum inflammatory factor level changes and disease severity in patients with chronic obstructive pulmonary disease complicated by tuberculosis. Int J Gen Med 18:3547–3556. doi:10.2147/IJGM.S522251 40607012 PMC12219164

[47] Kumar NP, Moideen K, Nancy A, Viswanathan V, Shruthi BS, Sivakumar S, Natarajan M, Kornfeld H, Babu S. 2019. Plasma chemokines are biomarkers of disease severity, higher bacterial burden and delayed sputum culture conversion in pulmonary tuberculosis. Sci Rep 9:18217. doi:10.1038/s41598-019-54803-w 31796883 PMC6890773

[48] Li X, Luo X, Wang B, Fu L, Chen X, Lu Y. 2024. Clofazimine inhibits innate immunity against Mycobacterium tuberculosis by NF-κB. mSphere 9:e0025424. doi:10.1128/msphere.00254-24 39046230 PMC11351037

[49] Qiang L, Wang J, Zhang Y, Ge P, Chai Q, Li B, Shi Y, Zhang L, Gao GF, Liu CH. 2019. Mycobacterium tuberculosis Mce2E suppresses the macrophage innate immune response and promotes epithelial cell proliferation. Cell Mol Immunol 16:380–391. doi:10.1038/s41423-018-0016-0 29572547 PMC6461940

[50] Li X, Luo X, Wang B, Fu L, Chen X, Lu Y. 2025. Sudapyridine (WX-081) inhibits Mycobacterium tuberculosis by targeting ATP synthase and upregulating host innate immunity. mSphere 10:e0014925. doi:10.1128/msphere.00149-25 40396746 PMC12188713

